# Psychometric Properties of the Malay Suicidal Behaviors Questionnaire-Revised (SBQ-R) in Malaysian Undergraduates

**DOI:** 10.3390/bs14111085

**Published:** 2024-11-12

**Authors:** Ching Sin Siau, Yee Kee Tan, Norhayati Ibrahim, Kairi Kõlves, Jie Zhang, Muhamad Nur Fariduddin, Bee Seok Chua, Whye Lian Cheah, Sharifah Munirah Syed Elias, Siti Nazilah Mat Ali, Serena In, Alex Lian Sheng Lim, Geetha Subramaniam, Walton Wider, Sherina Mohd Sidik, Siew Tin Tan, Bob Lew, Lai Fong Chan

**Affiliations:** 1 Centre for Community Health Studies (ReaCH), Faculty of Health Sciences, Universiti Kebangsaan Malaysia, Kuala Lumpur 50300, Malaysia; chingsin.siau@ukm.edu.my; 2 Centre for Healthy Aging and Wellness (H-Care), Faculty of Health Sciences, Universiti Kebangsaan Malaysia, Kuala Lumpur 50300, Malaysia; yatieibra@ukm.edu.my; 3 Institute of Islam Hadhari, Universiti Kebangsaan Malaysia, Bangi 43600, Malaysia; 4 Australian Institute for Suicide Research and Prevention, World Health Organization Collaborating Centre for Research and Training in Suicide Prevention, School of Applied Psychology, Griffith University, Mt. Gravatt, QLD 4122, Australia; k.kolves@griffith.edu.au (K.K.); boblew@asiacrux.com (B.L.); 5 School of Public Health, Shandong University, Jinan 250100, China; zhangj@buffalostate.edu; 6 Department of Sociology, State University of New York, Buffalo State, Buffalo, NY 14222, USA; 7 Department of Physical & Health Education, Faculty of Education, Universiti Teknologi MARA (Kampus Puncak Alam), Puncak Alam 42300, Malaysia; fariduddin@uitm.edu.my; 8 Psychology & Social Health Research Unit, Faculty of Psychology and Education, Universiti Malaysia Sabah, Kota Kinabalu 88400, Malaysia; chuabs@ums.edu.my; 9 Department of Community Medicine and Public Health, Faculty of Medicine and Health Sciences, Universiti Malaysia Sarawak, Kota Samarahan 94300, Malaysia; wlcheah@unimas.my; 10 Department of Special Care Nursing, Faculty of Nursing, International Islamic University Malaysia, Kuantan 25200, Malaysia; shmunirah@iium.edu.my; 11 Faculty of Business, Economics and Social Development, Universiti Malaysia Terengganu, Kuala Terengganu 21030, Malaysia; nazilah@umt.edu.my; 12 School of Psychology & Social Sciences, IMU University, Kuala Lumpur 57000, Malaysia; serenain@imu.edu.my; 13 School of Psychology, Quest International University, Ipoh 30250, Malaysia; alexlimls@outlook.com; 14 Faculty of Health and Life Sciences, INTI International University, Nilai 71800, Malaysia; geetha.subramaniam@newinti.edu.my; 15 Faculty of Business and Communications, INTI International University, Nilai 71800, Malaysia; walton.wider@newinti.edu.my; 16 Department of Applied Economic Sciences, Wekerle Sándor Üzleti Főiskola, 1083 Budapest, Hungary; 17 Faculty of Medicine and Health Sciences, Universiti Putra Malaysia, Serdang 43400, Malaysia; sherina@upm.edu.my; 18 Division of Nutrition & Dietetics, School of Health Sciences, IMU University, Kuala Lumpur 57000, Malaysia; siewtintan@imu.edu.my; 19 Department of Psychiatry, Faculty of Medicine, The National University of Malaysia (UKM), Cheras 56000, Malaysia; laifchan@hctm.ukm.edu.my

**Keywords:** psychometric, validation, suicidality, undergraduates, Malaysia

## Abstract

The psychometric properties of the Malay Suicidal Behaviors Questionnaire-Revised (SBQ-R) need to be tested as it is increasingly utilized, and there is a lack of a brief, validated scale to examine suicidal behaviors in Malaysia. A total of 713 and 715 undergraduates answered the English and Malay SBQ-R, respectively. Exploratory factor analysis derived a one-factor solution, with a total explained variance of 58.0% accounted for by the four items. Confirmatory factor analyses supported the one-factor model for the Malay SBQ-R, with acceptable fit indices (χ^2^/*df* = 0.451, comparative and Tucker–Lewis fit indices = ≥1.000, standardized root mean square residual = 0.014, root mean square error of approximation = 0.000, and 90% *CI* [0.000, 0.083]). Measurement invariance was achieved when comparing the SBQ-R between the English and Malay versions, indicating that both versions are similar in Malaysian undergraduates. Convergent validity was established through a strong correlation between the Malay SBQ-R and the Malay Yatt Suicide Attitude Scale (*r* = 0.74; *p* < 0.001). Good internal consistency was achieved for both the English (α = 0.83; ω = 0.85) and Malay (α = 0.81; ω = 0.84) versions. The Malay SBQ-R has adequate validity and reliability for use in Malaysian undergraduates.

## 1. Introduction

Suicide rates and behaviors are increasing in Malaysia. Based on an analysis of the World Health Organization Global Health Observatory data, from 2013 to 2019, there was a significant increase in age-standardized suicide rates [1]. The prevalence of suicidal ideation and suicide attempts among Malaysian secondary school adolescents was 13.1% and 9.5%, respectively, in 2022, compared with 7.9% and 6.8% in 2012 [2]. While efforts are mobilized to enact public health and individual suicide prevention measures, there is still a lack of a variety of assessment measures that could be employed in different public and clinical settings for the detection and assessment of suicidal ideation, attempts, and risk.

### 1.1. Overview of Suicidal Behavior Measurements in Malaysia

Suicidal behaviors in Malaysia have been studied extensively in various populations [3,4]. To facilitate establishing the levels and prevalence of suicidality, several instruments have been previously developed or validated in Malaysia. At the population level, Malaysia carries out the nationally representative National Health and Morbidity Survey (NHMS) on adult and adolescent populations. In the NHMS 2006, the General Health Survey was used to assess acute and chronic suicidal ideation [5]. The 2011 NHMS established suicidal ideation and suicide attempts using the WHO SUPREMISS Suicidality Questionnaire [6,7]. In the 2023 NHMS conducted among the adult population, item 9 of the PHQ-9 was used to establish recent suicidal ideation [8]. Among adolescents, the 2012, 2017, and 2022 NHMS Adolescent Health Survey employed questions from the Global School-Based Student Health Survey to determine past-12-month serious suicidal ideation, suicide plans, and suicide attempts [9]. While useful to determine prevalence, these instruments may be limited in describing nuances in suicidality, which are important for a greater understanding and intervention planning.

At the community level, longer scales are employed to measure a range of suicidal behaviors. The Yatt Suicide Attitude Scale (YSAS) is a 10-item instrument that was developed in the Malay language as a culturally appropriate measure that screens for suicidal ideation and suicide attempt risk in the Malaysian context [3]. With regard to translating and validating existing measures to assess suicidality, Sinniah et al. adapted the Positive and Negative Suicide Ideation (PANSI) Inventory into Malay with a multi-racial outpatient population in a Malaysian hospital to measure the level of suicidal ideation [10]. In addition, Sinniah and colleagues translated and validated the Reasons for Living Inventory in the Malaysian clinical population [11]. Furthermore, Halim et al. validated the Malay Revised Suicide Ideation Scale (R-SIS) among adolescents from a low-income housing area [12]. While these scales are helpful for the measurement of suicidal ideation and attempts, they could be relatively lengthy, with a range of 10 [3] to 35 items [11]. There is, therefore, a need for a brief scale that is valid for assessing suicidality in Malaysia.

### 1.2. Translation and Validation of the Suicidal Behaviors Questionnaire-Revised

The Suicidal Behaviors Questionnaire-Revised (SBQ-R) [13] is a four-item measure that has been employed widely in both clinical and general population settings to measure suicidal ideation, suicide plan, suicide attempt, communication of suicidal intent, future likelihood of suicidal behaviors, and general suicide risk. Several studies have validated the SBQ-R in various languages, including Polish among university students [14], Portuguese among adults [15], Spanish among nursing students [16] and patients with short-term suicide risk [17], Chinese among college students [18,19] and psychiatric patients [20], Iranian among undergraduates [21], and German among the general population [22].

A range of parameters were used to determine the validity of the SBQ-R across different countries and cultures. For example, in the Portuguese version [15], the criterion validity was established by calculating the ROC curve by comparing the SBQ-R scale score between suicide attempters and non-attempters. In the Spanish version, conducted among a clinical sample, the predictive validity was assessed by following up on the participants 30 days after discharge to determine the presence of suicide attempts or death [17]. Furthermore, the concurrent validity of the translated Spanish SBQ-R was demonstrated by examining its correlations with other psychological constructs of the reason for living and hopelessness [17]. In the Iranian version, the concurrent validity was determined through the correlation with single-item indices of suicide acceptability and lifetime suicidal ideation [21]. These measures were also used to test cut-off points of ≥7 and ≥8 in the Iranian SBQ-R [21]. In the German version, the convergent validity was assessed for depression, anxiety, and core constructs of the Interpersonal Theory of Suicidal Behavior scores [22]. The Chinese SBQ-R further tested the translated scale using the culture, comprehension, and translation bias procedure [18]. Several studies that conducted confirmatory factor analyses of the scale (the Polish [14], Portuguese [15], German [22], Spanish [17], and Iranian [21] versions) found a good fit for a one-factor solution. Exploratory structural equation modeling was used to examine the one-factor solution fit of the Chinese SBQ-R [18].

In Malaysia, the SBQ-R was forward- and backward-translated into Malay and was pilot-tested among public university medical students, revealing a good internal consistency reliability of α = 0.80 [23]. This measure was also used in studies across different populations, mainly university students [24,25] and older adults [26].

In light of the need for a brief scale to measure a range of suicidal behaviors and the lack of validity and reliability information on this existing scale, this study aimed to examine the psychometric properties of the Malay SBQ-R, specifically to examine (1) its factor structure, (2) convergent validity with the Malay YSAS, (3) internal consistency reliability, and (4) measurement invariance across the English and Malay language versions of the scale, and differences in terms of sex, ethnicity, and monthly household income.

## 2. Materials and Methods

### 2.1. Study Design

This was cross-sectional survey research in which university students were surveyed via online or pen-and-paper questionnaires across universities covering all regions in Malaysia. A cross-sectional study was feasible due to its ease of administration and low cost and is used extensively to examine various determinants of health, including suicidal behaviors [27].

### 2.2. Study Samples

Participants who were Malaysian, aged 18 and above, currently undergraduate students, and English- or Malay-literate were included. Those who refused to provide informed consent were excluded from this study. Undergraduate students were chosen due to past studies reporting a high prevalence of suicidal behaviors among them [24] and also for the relative availability of this population to be recruited for research purposes.

To conduct exploratory factor analysis (EFA), a minimum of 300 participants were recruited based on the rule of thumb from Tabachnick and Fidell [28]. For confirmatory factor analysis (CFA), the target number of participants was 200 [29], while for multi-group CFA (MGCFA), the present study aimed to have at least 100 participants in each group [30].

### 2.3. Data Collection

The undergraduate students from six public universities and three private universities in the northern, central, southern, and east coast regions of Peninsular Malaysia, as well as East Malaysia, were recruited using convenience sampling. The participants were approached by the researchers face-to-face or online. The survey was distributed via pen-and-paper format and a Google Form. Participants had the option of responding in the English or Malay language to the questionnaire. The researchers explained the purpose of this study to the participants and obtained their informed consent before they answered the survey. Mental health resources were embedded in the participant information sheet.

### 2.4. Measures

A set of questionnaires that consisted of several measures was utilized. To collect demographic information, questions about sex, age, year of study, ethnicity, religion, state of origin, and monthly household income were asked.

#### 2.4.1. The Suicidal Behaviors Questionnaire-Revised (SBQ-R)

The SBQ-R [13] is a brief self-report measure that consists of four items. The first item evaluates an individual’s lifetime suicidal ideation and lifetime suicide attempts, with responses ranging from 1 = Never to 4b = I have attempted to kill myself, and really hoped to die. The second item taps into the frequency of past-year suicidal ideation, where 1 = Never and 5 = Very often (5 or more times). The third item measures the communication of a suicide plan with a rating scale from 1 = No to 3b = Yes, more than once, and really wanted to do it. The last item assesses the likelihood of an individual attempting suicide in the future using a Likert scale point of 0 = Never to 6 = Very likely. The total score ranges from 3 to 18.

In this study, the α- and ω-coefficients indicated good internal consistency for the English and Malay SBQ-R (α = 0.83, 95% *CI* [0.81, 0.85] and α = 0.81, 95% *CI* [0.79, 0.83], respectively; ω = 0.85, 95% *CI* [0.82, 0.87] and ω = 0.84, 95% *CI* [0.81, 0.87], respectively).

#### 2.4.2. Yatt Suicide Attitude Scale (YSAS)

The YSAS was developed by Ibrahim et al. [3] within the Malaysian context. The scale is made up of two domains with five items, each measuring the risk of suicidal ideation and suicide attempts in the past two weeks. The items are rated on a 5-point Likert scale, where 1 = Never and 5 = Very often. The sum score ranges from 10 to 50, describing an individual’s overall suicide risk. Higher sum scores indicate a higher suicide risk. The sample items for each domain are: “Saya tidak ada keinginan untuk meneruskan kehidupan ini” (I have no will to continue my life; suicidal ideation risk); and “Saya pernah menggunakan kaedah tertentu untuk menamatkan hidup saya” (I have tried certain methods to end my life; suicide attempt risk).

The Malay version of the YSAS was validated among Malaysian university students [3] and showed good internal consistency (α = 0.84), convergent validity with the Suicide Ideation Scale (*r* = 0.38; *p* < 0.01) [31], and concurrent validity with Kessler’s K10 psychological distress scale (*r* = 0.64; *p* < 0.01) [32].

In this study, Cronbach’s alpha (α) demonstrated excellent internal consistency for the Malay YSAS (α = 0.93; 95% *CI* [0.93, 0.94]).

### 2.5. Data Analysis

The descriptive statistics analysis was carried out using IBM SPSS Statistics for Windows, version 27 [33]. The psych package [34] in the R software, version 4.2.0, was used to test the internal consistency of the scales (α- and ω-coefficients) and to conduct Spearman’s correlation analysis as well as exploratory factor analysis (EFA) using principal axis factoring. The boot package [35] was also used for bootstrapping purposes when computing the ω-coefficients. The lavaan [36] and semTools [37] packages were used for confirmatory factor analysis (CFA) and multigroup CFA (MGCFA).

The floor and ceiling effects of the scales were identified using the cut-off percentage suggested by McHorney et al. and Terwee et al. [38,39]. If the score distributions of the lowest or highest possible responses exceeded 15%, floor and ceiling effects were considered present, respectively. The descriptive statistics were calculated.

In terms of the convergent validity of the Malay SBQ-R in assessing suicide risk, Spearman’s correlation between the total scores of the Malay SBQ-R and the Malay YSAS should be greater than 0.50 for the total sample and the subsamples (see below) [40]. This scale was chosen because it also measures suicide risk in a university student population [13].

The total samples who responded to the Malay SBQ-R were randomly split into half by IBM SPSS Statistics to perform EFA (principal axis factoring) (*n* = 357) and CFA (*n* = 358). Promax rotation was adopted to explore the factor structure of the Malay SBQ-R. The Kaiser–Meyer–Olkin (KMO) test of sampling adequacy and Bartlett’s test of sphericity were used to examine the suitability of the data for factor analysis. Kaiser suggested that the KMO value should be >0.50 [41]. Bartlett’s test of sphericity should be significant (*p* < 0.05) to conduct a factor analysis [42]. Factors with an eigenvalue greater than one were retained [43]. In addition, the scree plot of eigenvalues was examined to determine the number of factors at the point where there was a break between the steep part of the slope (indicating factors with a large variance) and the gradual diminishing of the rest of the plot [44]. The communalities of the items should be >0.30 [45] to indicate that the items have an adequate proportion of variance explained by the extracted component. A factor loading of >0.50 was considered practically significant [43].

The fit indices used to evaluate the models in the CFA and MGCFA included the χ^2^/*df* ratio (<3.00) [46] with its *p*-value (>0.05) [47], CFI (≥0.95), Tucker–Lewis index (TLI ≥ 0.95), standardized root mean square residual (SRMR < 0.08), root mean square error of approximation (RMSEA < 0.06) [48], and 90% *CI*. The weighted least squares mean and variance-adjusted (WLSMV) estimation method was used in both the CFA and MGCFA.

The one-factor model of the SBQ-R was used to conduct the MGCFA to examine the measurement invariance between the English and Malay versions. First, the configural invariance was assessed via the overall model fit to identify if the SBQ-R had the same pattern of free and fixed loadings across both languages. Later, the metric invariance was assessed to determine if the items in the English and Malay versions contributed to the latent construct to a similar extent. The scalar invariance was assessed to examine the equivalence of the item intercepts across the languages [49]. A change value of less than 0.01 in the CFI (∆ CFI) between the models supported the measurement invariance [50]. Lastly, the residual invariance was assessed to determine the equivalence of the item residuals of the metric and scalar invariant items. Residual invariance was supported if the overall fit of the residual invariance model was not significantly worse than the scalar invariance model [49]. Based on the Malay version of the questionnaire, independent-sample *t*-tests and one-way ANOVA were used to test whether there were mean differences between sex, ethnicity, and monthly household income. *p*-values of <0.05 (two-tailed) were considered significant.

## 3. Results

### 3.1. Descriptive Statistics

Prior to the data analysis, cases with missing values (*N* = 9), yea–nay responses [51] (*N* = 8), and those that did not fulfill the criteria for nationality (*N* = 12) were removed listwise. A total of 1428 participants (*N*_English_ = 713 and *N*_Malay_ = 715) were retained for further analysis. Most of the participants were female (69.6%), year 1 undergraduate students (33.4%), Malay (43.3%), Muslim (48.4%), from the state of Selangor (16.2%), and with a monthly household income of MYR 4,850 and below (54.5%).

The mean scores of the Malay SBQ-R (total sample, subsample 1, and subsample 2) and YSAS based on the total scores are presented in Table 1. The range of the Malay SBQ-R scale scores is also provided for the total sample and subsamples (Table 1). Floor effects were identified for the total scores of the Malay SBQ-R (56.1%), English SBQ-R (50.1%), and Malay YSAS (52.6%). However, no ceiling effect was detected for any of the scales.

Item-level descriptive analysis of each item in the Malay SBQ-R was conducted, and the results are reflected in Table 2. The results based on item 1 show that 22.5% had lifetime passive suicidal ideation, 11.3% had lifetime suicide plans, and 2.7% had lifetime suicide attempts. Based on item 2, 33.1% had prior 12-month suicidal ideation. A large majority (82.1%) had never communicated with others about suicidal ideation, plans, or attempts, based on item 3. Item 4 shows that 5.1% thought they were likely, rather likely, or very likely to attempt suicide one day.

### 3.2. Construct Validity of the Malay SBQ-R

**Figure 1:**
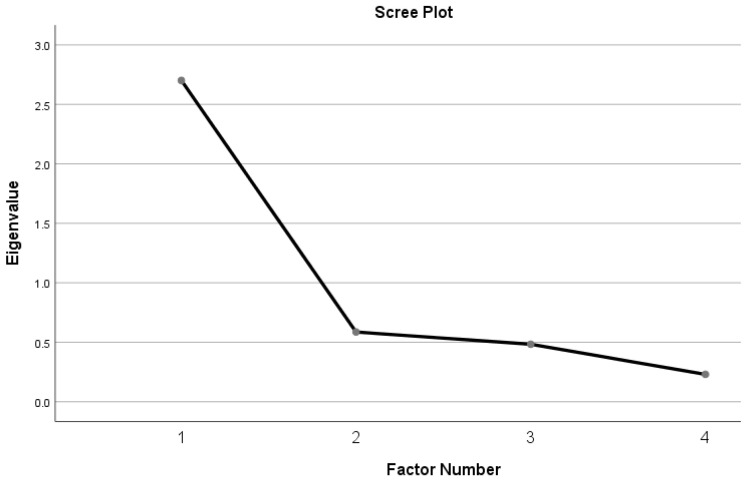
Scree plot of the exploratory factor analysis.

The KMO test for sampling adequacy (0.78) and Bartlett’s test of sphericity (χ^2^[6] = 615.18; *p* < 0.001) demonstrated the suitability of the data for factor analysis. The communalities for each item ranged from 0.36 to 0.78. The factor loadings ranged from 0.60 to 0.88. All items were retained. Only one factor showed an eigenvalue of greater than one. Examination of the scree plot showed that after the first factor, there was a flattening of the plot from the second factor onwards (see Figure 1). The total variance explained by the factor was 58.0% (Table 3).

The findings from the CFA show that all the model fit indices for the Malay SBQ-R one-factor model were satisfactory (refer to model 1 in Table 4). The standardized factor loadings for items 1 to 4 in the CFA were 0.92, 0.95, 0.77, and 0.78, respectively. The same model was then tested across the grouping variable of language (i.e., English and Malay versions). The cut-off values for all model fit indices were met in model 2 (Table 4), indicating that configural invariance was achieved. The differences in the CFI values between models 2 and 3 (0.000), as well as models 3 and 4 (0.001), were less than 0.01; therefore, measurement invariance was supported. The model fit indices for models 4 and 5 were similar, demonstrating that residual invariance was achieved.

A comparison of the mean scores between sex, ethnicity, and monthly household income found that there was a mean difference in sex (*t* (224.35) = −3.20; *p* < 0.001; Cohen’s *d* = 0.44), where females had higher scores than males. However, comparisons between ethnicity (*F* (2, 355) = 0.90; *p* = 0.480; Cohen’s *f* = 0.123) and monthly household income (*F* (2, 355) = 1.07; *p* = 0.345; Cohen’s *f* = 0.071) showed non-significant results.

### 3.3. Convergent Validity of the Malay SBQ-R

Due to the non-parametric data in the Malay YSAS, Spearman’s correlation analysis was used to examine the strength of the association between the Malay SBQ-R and Malay YSAS (see Supplementary Materials). Spearman’s correlation coefficient showed that the total scores of the Malay SBQ-R and Malay YSAS were positively correlated (r = 0.74; *p* < 0.001), sufficient to indicate the convergent validity of the Malay SBQ-R. In addition, the correlation between the Malay SBQ-R in sub-sample 1, sub-sample 2, and the YSAS was also significant (*r* = 0.72, *p* < 0.001 and *r* = 0.76, *p* < 0.001).

## 4. Discussion

Due to the need for a brief instrument to measure a range of suicidal behaviors in Malaysia, this study was conducted to examine the psychometric properties of the Malay SBQ-R. The results from exploratory and confirmatory factor analyses showed a one-factor solution to have adequate psychometric vigor in its factor structure. A one-factor structure was also found in the original SBQ-R [13], as well as translated versions in Asia, such as the Chinese [18] and Iranian [21] versions. This shows suicidal behaviors are perceived as a single construct in Malaysian undergraduates.

Multi-group CFA showed that the SBQ-R was comparable between the Malay and English versions. This is important as the SBQ-R has been used in the English (e.g., [25]) and Malay [23] languages across studies in Malaysia. Establishing the measurement invariance across these languages showed that the SBQ-R in Malay and English are comprehended similarly when administered in English or Malay to Malaysian undergraduates. This may be due to English being a second language in Malaysia, and undergraduates need to fulfill English and Malay language requirements prior to acceptance into a higher learning institution in Malaysia, thus ensuring their knowledge of the two languages is on par. Additionally, a strong correlation between the Malay SBQ-R and Malay YSAS established its convergent validity. This convergence with the YSAS is important as the YSAS was developed and validated in Malay, thus suggesting the Malay SBQ-R’s suitability for administration in the Malaysian context.

With regard to the internal consistency of the Malay SBQ-R, the α- and ω-coefficients of more than 0.80 indicated good reliability and were similar to the α-coefficient obtained in Tan et al.’s study [23]. This shows the Malay SBQ-R measures a construct of suicidal behaviors that includes all items pertaining to suicidal ideation, plans and attempts, the communication of suicidal behaviors, and the self-perceived future probability of engaging in suicidal behaviors [52]. This supports the use of a single total score of the Malay SBQ-R to indicate the level of suicide risk or behaviors.

The validation of the SBQ-R enables its practical application in measuring suicidality in the general population setting among Malaysian university students. As a population with high suicidality [53], the validation of this measure enables more robust studies among university students in the future, both in determining the overall suicide risk severity and various suicidal behavior parameters enabled by item-level analysis of the scale. The Malay SBQ-R is also practical in its brevity compared with other existing measures, in addition to being available for use free of charge. A longer scale may have incurred an increased burden on individuals who may already be distressed. However, there are relative shortcomings to using this short and unidimensional scale in determining suicide risk levels. First of all, it is not within the purpose of the SBQ-R to measure qualitative variations within types of suicidal ideation, planning, and attempts (e.g., positive and negative suicidal ideation domains as measured by the PANSI). Therefore, it is best to utilize the SBQ-R in addition to other scales and to follow up with other methods of investigation, such as diagnostic or semi-structured in-depth interviews, to meet the requirements of assessing suicidality within clinical, therapeutic, or research contexts.

This study had a few strengths and limitations. All regions in Malaysia (the northern, central, southern, and east coast regions of Peninsular Malaysia and East Malaysia) were represented in this study. However, using the convenience sampling method to recruit the students may have introduced bias. The cross-sectional nature of this study precludes testing the predictive utility of this scale. The sample information for the field of study, an important consideration for university students, was not collected, and this should be performed in future studies. In addition, utilizing a university student sample has limitations in its homogeneity (e.g., a narrow age range and similar educational backgrounds) and non-representativeness. Future studies could benefit from sampling from the general population (including both younger and older populations) and from clinical settings to enable inferences to be made in these populations as well. In addition, there are shortcomings in the included measurement tool for assessing validity, namely, the Malay Yatt Suicide Attitude Scale, which lacks a cut-off point in its Malay version, precluding the possibility of using it as a tool for establishing criterion validity. Establishing a cut-off score for suicide risk may require further validation in a clinical sample, with a focus on its predictive ability to ascertain suicidal ideation and attempt cases. Still, suicidality is difficult to predict [54]. Finally, there was also a lack of re-confirmation of the factor structure in a subsequent study.

In conclusion, this study found that the Malay SBQ-R has adequate psychometric vigor for use among undergraduate students in Malaysia. Its one-factor solution is consistent across other studies, and adequate internal consistency reliability indicated that all items measured the same construct. The Malay and English versions of the questionnaire were comparable through the establishment of their measurement invariance. Future studies could examine the predictive ability of the Malay SBQ-R in identifying suicidal ideation and attempts in clinical samples.

## Figures and Tables

**Table 1:** Demographic characteristics and suicidality scores of participants for the total sample (*N* = 1428).

Variable	Frequency (%)
Age (mean ± *SD*)	21.55 ± 1.63
Language used in survey	
English	713 (49.9)
Malay	715 (50.1)
Sex	
Male	434 (30.4)
Female	994 (69.6)
Year of study	
Year 1	477 (33.4)
Year 2	426 (29.8)
Year 3	448 (31.4)
Year 4	61 (4.3)
Year 5	16 (1.1)
Ethnicity	
Malay	619 (43.3)
Chinese	486 (34.0)
Indian	130 (9.2)
Bumiputera Sabah	92 (6.5)
Bumiputera Sarawak	79 (5.5)
Others	22 (1.5)
Religion	
Islam	691 (48.4)
Buddhism/Daoism	370 (25.9)
Hinduism	115 (8.1)
Christian	192 (13.4)
Others	29 (2.0)
No religious affiliation	31 (2.2)
State of origin	
Perlis	6 (0.4)
Kedah	58 (4.1)
Penang	43 (3.0)
Perak	223 (15.6)
Selangor	232 (16.2)
Negeri Sembilan	68 (4.8)
Melaka	22 (1.5)
Johor	158 (11.1)
Kelantan	115 (8.0)
Terengganu	75 (5.3)
Pahang	54 (3.8)
Sabah	127 (8.9)
Sarawak	156 (10.9)
Federal Territories (Kuala Lumpur, Putrajaya, and Labuan)	90 (6.3)
Not stated	1 (0.1)
Monthly household income	
MYR 4850 and below	778 (54.5)
MYR 4851–MYR 10,970	456 (31.9)
MYR 10,971 and above	194 (13.6)
Total score of the Malay SBQ-R for the total sample (mean ± *SD*); range	4.82 ± 2.86 3, 18
Total score of the Malay SBQ-R for subsample 1 (mean ± *SD*); range	4.78 ± 2.83 3, 17
Total score of the Malay SBQ-R for subsample 2 (mean ± *SD*); range	4.85 ± 2.90; 3, 18
Total score of the English SBQ-R (mean ± *SD*); range	5.00 ± 2.94; 3, 18
Total score of the Malay YSAS for the total sample (mean ± *SD*); range	13.30 ± 5.92; 10, 48

Note: *SD* = standard deviation; SBQ-R = Suicidal Behaviors Questionnaire-Revised; YSAS = Yatt Suicide Attitude Scale.

**Table 2:** Item-level analysis of each item in the Malay Suicidal Behaviors Questionnaire-Revised for the total sample (*N* = 1428).

Item No.	Item	Frequency Answering Yes (*n*)	Percentage (%)
1	Pernahkah anda berfikir atau cuba membunuh diri anda? (Have you ever thought about or attempted to kill yourself?)		
	Option 1: Tidak pernah. (Never)	454	63.5
	Option 2: Fikiran itu sepintas lalu sahaja. (It was just a brief passing thought)	161	22.5
	Option 3a: Saya ada sekurang-kurangnya sekali rancangan untuk membunuh diri, tetapi tidak cuba untuk melakukannya. (I have had a plan at least once to kill myself but did not try to do it.)	66	9.2
	Option 3b: Saya ada sekurng-kurangnya sekali rancangan untuk membunuh diri, dan saya betul-betul nak mati. (I have had a plan at least once to kill myself and really wanted to die.)	15	2.1
	Option 4a: Saya pernah cuba membunuh diri, tetapi saya tak nak mati. (I have attempted to kill myself, but did not want to die.)	15	2.1
	Option 4b: Saya pernah cuba membunuh diri, dan saya betul-betul berharap saya akan mati. (I have attempted to kill myself, and really hoped to die.)	4	0.6
2	Berapa kerap anda berfikir tentang membunuh diri dalam tahun yang lepas? (How often have you thought about killing yourself in the past year?)		
	Option 1: Tidak pernah (Never)	478	66.9
	Option 2: Jarang (1 kali) (Rarely (1 time))	117	16.4
	Option 3: Kadang kala (2 kali) (Sometimes (2 times))	76	10.6
	Option 4: Kerap (3–4 kali) (Often (3-4 times))	27	3.8
	Option 5: Sangat kerap (5 atau lebih) (Very often (5 or more times))	17	2.4
3	Pernahkah anda memberitahu sesiapa bahawa anda hendak membunuh diri, atau anda mungkin berbuat demikian? (Have you ever told someone that you were going to commit suicide, or that you might do it?)		
	Option 1: Tidak (No)	587	82.1
	Option 2a: Ya, sekali, tetapi betul-betul tak nak mati (Yes, at one time, but did not really want to die)	92	12.9
	Option 2b: Ya, sekali, dan betul-betul nak berbuat demikian (Yes, at one time, and really wanted to die)	13	1.8
	Option 3a: Ya, lebih daripada sekali, tetapi tak nak berbuat demikian (Yes, more than once, but did not want to do it)	20	2.8
	Option 3b: Ya, lebih daripada sekali, dan betul-betul nak berbuat demikian (Yes, more than once, and really wanted to do it)	3	0.4
4	Apakah kemungkinan yang anda akan cuba membunuh diri pada sesuatu hari? (How likely is it that you will attempt suicide someday?)		
	Option 0: Tidak pernah (Never)	581	81.3
	Option 1: Tidak ada peluang langsung (No chance at all)	30	4.2
	Option 2: Agak tidak mungkin (Rather unlikely)	44	6.2
	Option 3: Tidak mungkin (Unlikely)	23	3.2
	Option 3: Mungkin (Likely)	23	3.2
	Option 4: Agak mungkin (Rather likely)	11	1.5
	Option 5: Kemungkinan besar (Very likely)	3	0.4

**Table 3:** Exploratory factor analysis of the Malay Suicidal Behaviors Questionnaire-Revised (*n* = 357).

Item No.	Item	Factor Loadings	Communalities
Total variance explained		0.58	
1	Pernahkah anda berfikir atau cuba membunuh diri anda? (Have you ever thought about or attempted to kill yourself?)	0.85	0.73
2	Berapa kerap anda berfikir tentang membunuh diri dalam tahun yang lepas? (How often have you thought about killing yourself in the past year?)	0.88	0.78
3	Pernahkah anda memberitahu sesiapa bahawa anda hendak membunuh diri, atau anda mungkin berbuat demikian? (Have you ever told someone that you were going to commit suicide, or that you might do it?)	0.60	0.36
4	Apakah kemungkinan yang anda akan cuba membunuh diri pada sesuatu hari? (How likely is it that you will attempt suicide someday?)	0.67	0.45

**Table 4:** Confirmatory factor analysis (CFA) using the one-factor model of the Malay SBQ-R and multi-group CFA comparing the English and Malay SBQ-R (*n* = 358).

	χ^2^ (*df*)	χ^2^/*df*	CFI	TLI	SRMR	RMSEA [90% *CI*]
Model 1	0.901(2)	0.451	1.000	1.001	0.014	0.000 [0.000, 0.083]
Model 2	0.400(4)	0.100	1.000	1.001	0.005	0.000 [0.000, 0.000]
Model 3	4.839(7)	0.691	1.000	1.000	0.015	0.000 [0.000, 0.036]
Model 4	31.857(17)	1.874	0.999	0.999	0.009	0.035 [0.015, 0.054]
Model 5	31.857(17)	1.874	0.999	0.999	0.009	0.035 [0.015, 0.054]

Note: χ^2^ = chi-square; *df* = degree of freedom; CFI = comparative fit index; TLI = Tucker–Lewis index; SRMR = standardized root mean square residual; RMSEA = root mean square error of approximation; model 1 = one-factor model in CFA; model 2 = configural invariance model in MGCFA; model 3 = metric invariance model in MGCFA; model 4 = scalar invariance model in MGCFA; model 5 = residual invariance model in MGCFA. The *p*-values for the chi-square test were non-significant for models 1, 2, and 3.

## Data Availability

The original contributions presented in this study are included in the Supplementary Materials. Further inquiries can be directed to the corresponding author.

## Supplementary Materials

The following supporting information can be downloaded at: https://www.mdpi.com/article/10.3390/bs14111085/s1, File S1: Malay SBQ-R and YSAS Dataset.
